# A Role for Ultrasonic Vocalisation in Social Communication and Divergence of Natural Populations of the House Mouse (*Mus musculus domesticus*)

**DOI:** 10.1371/journal.pone.0097244

**Published:** 2014-05-09

**Authors:** Sophie von Merten, Svenja Hoier, Christine Pfeifle, Diethard Tautz

**Affiliations:** Department Evolutionary Genetics, Max Planck Institute for Evolutionary Biology, Plön, Germany; University of Missouri, United States of America

## Abstract

It has long been known that rodents emit signals in the ultrasonic range, but their role in social communication and mating is still under active exploration. While inbred strains of house mice have emerged as a favourite model to study ultrasonic vocalisation (USV) patterns, studies in wild animals and natural situations are still rare. We focus here on two wild derived mouse populations. We recorded them in dyadic encounters for extended periods of time to assess possible roles of USVs and their divergence between allopatric populations. We have analysed song frequency and duration, as well as spectral features of songs and syllables. We show that the populations have indeed diverged in several of these aspects and that USV patterns emitted in a mating context differ from those emitted in same sex encounters. We find that females vocalize not less, in encounters with another female even more than males. This implies that the current focus of USVs being emitted mainly by males within the mating context needs to be reconsidered. Using a statistical syntax analysis we find complex temporal sequencing patterns that could suggest that the syntax conveys meaningful information to the receivers. We conclude that wild mice use USV for complex social interactions and that USV patterns can diverge fast between populations.

## Introduction

House mice (*Mus musculus*) are known to emit ultrasonic vocalisations (USV) in many social contexts. Mouse pups utter USV when cold or separated from their mother [1] and adolescent mice use USV in social interaction with each other [2]. Mature male mice emit USV with song characteristics in mating contexts such as stimulation through odour cues from females [3]–[6]. Most USV studies have so far been conducted with inbred mouse strains. It was shown that male and female mice emit USV [7], [8] and that this plays an important role in mate attraction and selection [5], [9], [10]. Using knockout mice for hearing ability [11] or cross-fostering experiments [12] showed that general USV characteristics are genetically inherited, not learned. On the other hand, auditory feedback may nevertheless be necessary to maintain certain ultrasonic song features [13] and behavioural preferences [14]. The question if mice perform some way of vocal learning is currently being investigated and discussed [15].

The classic example for acoustic sexual signalling is the song of passerine birds. In these birds, one or both sexes emit species specific songs to convey the ownership of a territory and to attract possible mates [16]. Not only birds use vocalisation within the mating context, but many species from all other groups of vertebrates as well [17], [18]. Songs emitted in a mating context do not only convey species membership, but can also hint at the reproductive status [19], fitness [20] and individuality [21] of the singer. However, interactions through vocalisation can be important beyond the mating situation and are thought to serve additional roles in the maintenance of complex communities [22]–[24].

In a previous study using mice from two natural populations from France and Germany, we found assortative patterns of mate choice according to their population origin [25]. Although it is known that olfactory cues play a major role in mate choice in mice [26] we speculated that USV divergence is an alternative mechanism to cause this differential population recognition. Although the two populations separated less than 3,000 years ago, genome scans revealed several hundred molecularly highly differentiated regions between them, indicating adaptive divergence [27]–[29]. Intriguingly, one of these regions (see suppl. Figure S1) is found in *Cntnap2*, a target gene of *FoxP2*. *FoxP2* is a well studied transcription factor that regulates a pathway which has been implicated in human speech and language disorders [30] as well as in song specification in birds and other animals [31]. Given these molecular hints, it seems indeed possible that USV between the populations may have diverged in a way that allows differential recognition.

In the current study we have therefore assessed acoustic and syntactic differences in the song of wild house mice from the French and the German population. We use an experimental setup that allows not only to assess differences between populations, but also between sexes and in different dyadic social contexts. Our results provide evidence for USV pattern divergence between the populations and give further proof that vocalisation is not only used in mating situations, but also in other social interactions such as encounters between females.

## Methods

### Ethics statement

The animals used in this study are *Mus musculus*, a species that is not protected. Permits for catching them were not required at the time they were caught. Some specimens were caught on the properties of private landowners, with their oral permission to enter the property and catch mice. Mice were trapped in live traps, provided with food and shelter, by experienced personnel under the direction of DT. Trapping was only conducted at moderate temperature conditions, so that there was no danger for trapped animals to suffer from heat or cold. After trapping, mice were transferred into standard mouse cages containing food, water and shelter. Transportation to the laboratory, maintenance and handling were conducted in accordance with German animal welfare law (Tierschutzgesetz) and FELASA guidelines. Permits for keeping mice were obtained from the local veterinary office “Veterinäramt Kreis Plön” (permit number: 1401-144/PLÖ-004697).

### Study species, breeding and housing

The studied mice were derived from wild caught mice from two populations: one originating from Southern France (Massif Central region), the other from Western Germany (Cologne/Bonn region). Mice were caught in 2005 in France and in 2006 in Germany. The sampling scheme was designed to obtain a representative set of mice from the respective populations and to avoid trapping related individuals [28]. Mice were kept in the mouse facility of the Max Planck Institute for Evolutionary Biology in Plön, Germany.

We applied a rotating outbreeding design [32] with 10 unrelated starting pairs, which ensures a maximum degree of outbreeding [33]. Breeding pairs were kept for one or two litters, with parents and offspring housed together up to the weaning of the young at ∼30 days of age. After weaning, some mice were kept individually, some stayed in equal sex-groups with siblings, depending on their mutual compatibility. In preparation for the experiments, all mice were held in individual cages inside the experimental room.

All mice were kept in standard lab cages (Type II and III, Bioscape, Germany). In addition to standard bedding (Rettenmaier, Germany) we provided enrichment (paper stripes, wood wool, a cardboard box and a spinning wheel (Plexx, Netherland)) in each cage. Food (Standard Diet 1324, Altromin, Germany) and water was provided *ad libitum*. Experimental and keeping rooms were climate controlled (20–24°C, 50–65% humidity) and maintained on a 12∶12 light-dark schedule with lights on at 7 am.

We recorded from 19 mature females (9 French and 10 German) and 18 mature males (8 French and 10 German) from the F3 to F5 progeny of the wild caught mice. 6 German females, 6 German males, 7 French females, and 8 French males were recorded in all three social contexts (see Methods section *Recording schedule*), and these were used for statistical analysis. Before and after preparation and testing period, all experimental mice were housed in the colony, sharing rooms with the breeding populations.

### Sound recordings

**Figure 1:**
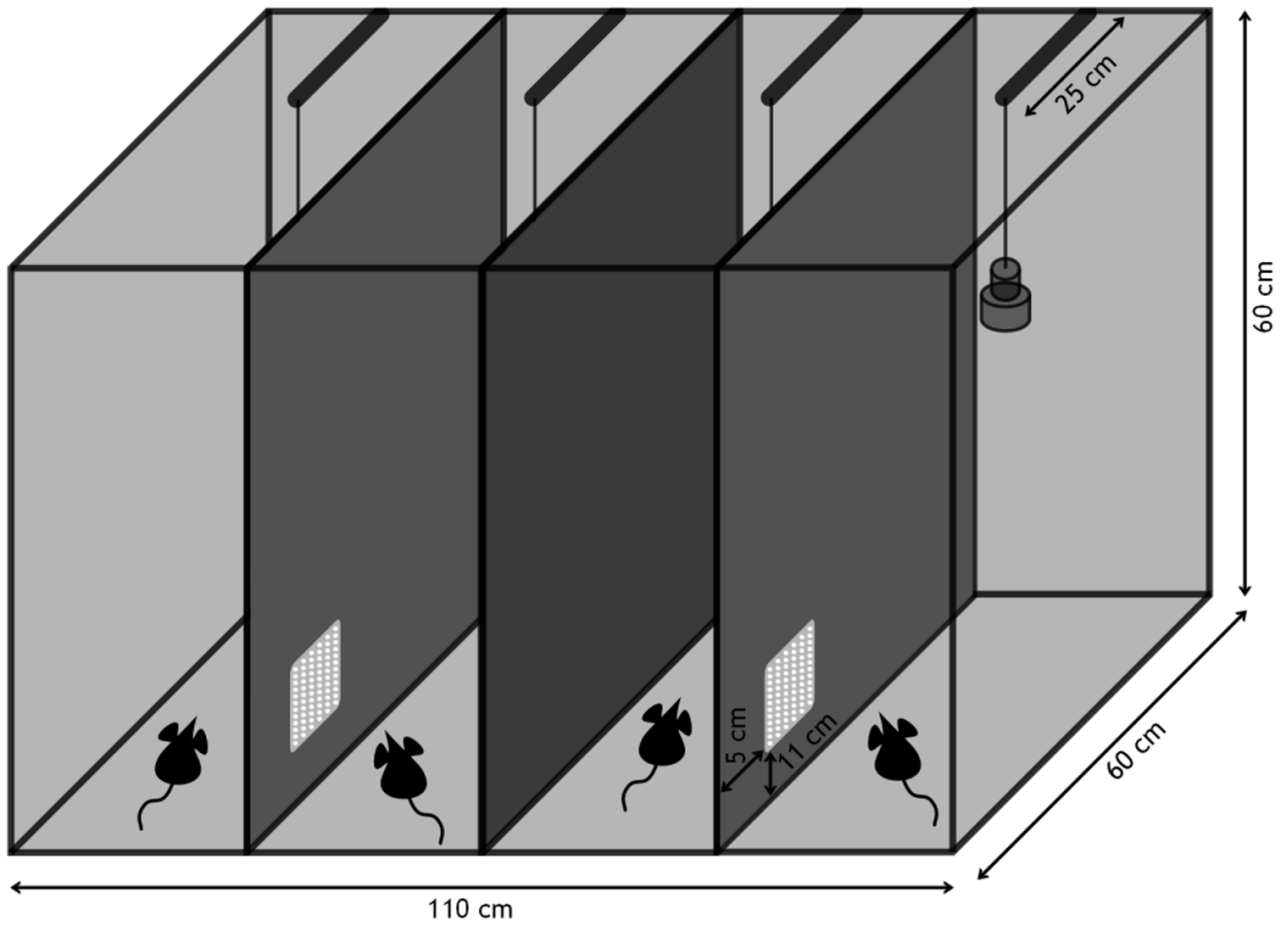
Scheme of the USV recording box. The box is made from grey PVC (side and back walls) and non-reflecting glass (front window). Four equal compartments are equipped with bedding, food and water, and acoustically monitored via an ultrasound microphone from above. The partner compartments (compartment pairs at the left and right) are connected via a little window made from metal grid (indicated in white) to allow sensory contact between recording partners (drawing not to scale.)

Sound recordings were conducted in a separate room (20–24°C, 35–55% humidity) inside a USV recording box (Figure 1). The recording box was custom-built from grey PVC (side walls and floor), metal grid (top) and non-reflecting glass (front). It consisted of four separate compartments, each measuring 60×25×60 cm (l×w×h). The two left and the two right compartments were connected via a window made from a perforated metal plate (dimension of window 5×5 cm, spacing of the metal plate 1 mm). This window could be tightly closed by attaching a fitting piece of PVC. With the window open, the two mice sitting in such two neighbouring compartments (termed "recording partners" in the following) had the chance to use it for visual, olfactory, acoustic and partly tactile contact. Each compartment was equipped with standard bedding material, paper stripes and a cardboard box. Food and water was provided *at libitum*.

Each compartment was fitted with one ultrasound-microphone (30 cm above ground, 25 cm distance from back wall; condenser ultrasound microphone CM16/CMPA, Avisoft Bioacoustics, Germany). All four microphones were connected to a multi-channel recording device (Avisoft UltraSoundGate 416, 4-channel). Recordings were made with a sampling rate of 250 kHz and a depth of 8 bit (software: Avisoft USG Recorder). We used the “whistle tracking” option, to automatically detect mouse USV. To trigger a recording, a USV ranging from 20–250 kHz had to last at least 10 ms. Once started, a recording event lasted until 1 second after the last automatically detected whistle. Further, a pre-trigger of 200 ms was applied to not miss the beginning of a USV element.

The microphones were attached in the given position (slightly away from the contact window) to minimize the recording of the USV emitted by the recording partner. To further reduce the possibility to take the USV of a recording partner as the USV of the mouse of interest, we did not use any USV recordings that were simultaneously recorded in more than one of the compartments. Respective recordings were removed semi-automatically (scripts written by Bernhard Haubold, Max Planck Institute).

To estimate the audibility of mouse USV from a recording partner compartment, as opposed to a non-partner compartment, we measured the amplitude of mouse USV played back at natural amplitudes from the partner compartment with open and with closed window. The amplitude of the re-recorded USV of an 80 kHz sound was 68 dBSPL and 26 dBSPL with open and closed window, respectively. Considering the hearing threshold of mice (around 60 dBSPL at 80 kHz, [34]), it is highly unlikely that mice were able to perceive the USV of other mice sitting in a non-partner compartment, or mice sitting in the partner-compartment with closed contact-window.

In the experimental room, we also kept the other mice of the same recording group waiting for their turn to be recorded. The keeping rack was at a distance of 3–4 m away from the recording box. This added to the general olfactory impression of the room, but from the keeping facilities mice were well used to such olfactory impressions. We also deem the potential influence of acoustic disturbance as highly unlikely: Ultrasound has a high natural attenuation, e.g. frequencies of 80 kHz will be attenuated by approximately 2.5 dB/m at the given temperature and humidity [35]. Given the 3–5 m distance of the caged mice to the experimental mice, USV emitted by the caged mice would arrive at the floor of the recording compartments reduced by at least 7.5–12.5 dB not even taking any obstacles (walls of cages or recording box) into account. Considering the hearing threshold of mice (see above), it is unlikely a mouse could perceive the USV of the other mice living in the room. Sound in the sonic range travels further than ultrasound. Thus it is likely that the sounds of other mice squeeking or moving (e.g. in the spinning wheel) reach the mice in the recording boxes. Like the olfactory impressions mentioned above, such sounds are a typical part of the environment of socially living house mice. Likewise, our mice were used to these sounds from the keeping colonies.

### Recording schedule

Each recording session lasted four days. At the beginning of each recording session, four mice were placed individually in the four cleaned and freshly furbished compartments. The contact windows between partner compartments were closed. The mice were given an accommodation period of two days and nights to get familiar with their new environment. Before the onset of the third night, the contact windows were opened to allow sensory contact between recording partners. The recordings for this study were conducted during the two nights after opening the contact window (nights three and four), each night beginning at lights-off (7 pm) and ending at lights-on (7 am). The two recording nights offered us the possibility to differentiate between two levels of social familiarity: less familiar in the first, more familiar in the second recording night.

Recording partners were chosen according to the social context: (1) Different sex, different population (DiffPop): the recording partners were of different sex and different population; (2) Different sex, same population (DiffSex): the recording partners were of different sex but the same population; (3) Same sex, same population (SameSex): the recording partners were of the same sex and the same population. We did not control for the oestrus stage of females for several reasons. First, several previous studies did not find an influence of female oestrus stage on male USV responses [5], [10]. In one recent study [36] such an influence was found. In this study, females in pro-oestrus seemed to evoke male USV syllables with low dominant frequencies, long duration and high bandwidth, while for females in di-oestrus it were syllables with high dominant frequencies, short duration and low bandwidth; for females in oestrus syllables were intermediate in all parameters. However, in this study it was not checked whether only males or also females were emitting USV. The observed differences in USV parameters might indeed result from males or females changing the structure of their USV according to the different stages of oestrus. It can, however, not be ruled out that the observed differences resulted from males and females changing the amount of emitted USV reciprocally, i.e. males singing more and females less, or the other way round. As the structure of USV is likely sex-specific (see results and discussion of this study) such a reciprocal change in amount of USV would lead to a perceived structural change of USV, if males and females are recorded together. Secondly, oestrus has to be measured every day at least once [36]. This puts the females into stress, which in turn can influence their vocalisation behaviour. As we recorded for two consecutive days, females were likely passing through different phases of oestrus during our experiments.

### Sound analysis

We analysed the number of songs emitted per mouse in each social context and night. We further analysed several temporal and spectral features of the songs and syllables to compare between different populations and sexes, and according to the social contexts and familiarity. A syllable is defined as a single USV-element, separated from other single USV-elements by at least 55 ms. A song is defined as bouts of such syllables, separated from other such bouts by at least 500 ms. We chose the respective time intervals after visual inspection of syllables and songs by two researchers experienced with mouse USV.

We conducted a detailed spectrographic analysis of the first 30 songs emitted by each individual mouse during each recording night. This number was chosen after a bootstrapping analysis (custom Matlab routine by SVM, Matlab R2012a, The MathWorks, USA), comparing the variance in song parameters for the analysis of the first 6, 12, 18, 24, 30, or 36 songs, respectively. This variance increased steeply up to 30 songs included and then levelled out.

The spectrographic analysis of syllables was conducted in three steps: (1) we extracted the frequency–time course of each syllable over time (further detailed below), (2) we calculated several temporal and spectral parameters (see Table 1) and (3) we conducted the statistical analyses.

**Table 1:** All temporal and spectral parameters used in the main analysis. For each group of mice the mean (+/− standard deviation) is given.

	German females	German males	French females	French males
Quantitative parameter set 1: number of songs				
songs/night	20.3 (+/−14.5)	12.53 (+/−13.74)	17.2 (+/−14.3)	14.6 (+/−45.8)
Quantitative parameter set 2: temporal data				
song duration	505.9 (+/−382.9)	300.3 (+/−181.0)	565.3 (+/−479.4)	294.0 (+/−183.4)
syllables/second	20.0 (+/−17.6)	29.25 (+/−18.2)	25.6 (+/−14.5)	31.8 (+/−20.9)
Qualitative parameter set: syllable data				
duration	69.4 (+/−36.5)	46.3 (+/−36.5)	50.6 (+/−36.4)	36.5 (+/−26.0)
freq_sta_ (1)	78.4 (+/−8.2)	80.1 (+/−14.2)	76.3 (+/−10.8)	81.5 (+/−18.6)
freq_slope_ (1)	0.1 (+/−0.3)	0.2 (+/−0.5)	0.1 (+/−0.5)	0.1 (+/−0.6)
freq_min_ (1)	68.6 (+/−11.4)	75.0 (+/−14.0)	65.3 (+/−11.1)	75.5 (+/−18.5)
freq_band_ (1)	23.0 (+/−14.4)	14.5 (+/−10.6)	22.9 (+/−14.3)	15.1 (+/−14.4)
freq_COG_ (1)	78.1 (+/−10.6)	82.6 (+/−13.3)	75.6 (+/−9.8)	83.6 (+/−18.8)
jumps	0.6 (+/−0.9)	0.1 (+/−0.4)	0.8 (+/−1.1)	0.2 (+/−0.5)
turns	2.0 (+/−1.8)	1.2 (+/−1.5)	2.0 (+/−2.2)	0.9 (+/−1.1)

(1) start frequency, frequency slope (calculated as change of frequency in kHz per ms), minimum frequency, frequency band (calculated as change of frequency in kHz per ms) and COG of frequency (COG = Centre of gravity, see Methods section *Sound analysis* for calculation).

To extract the frequency–time course of USVs, we displayed the recordings as colour spectrograms using a 256 kHz Fast Fourier Transformation (FFT, Hann window; software: Selena, Department of Animal Physiology, University of Tübingen; Germany). Temporal reading accuracy was improved by FFT overlap (85%) to 0.002 ms; spectral reading accuracy was improved by zero-padding to 0.49 kHz. The frequency-time course of each syllable was tracked semi-automatically by the software by selecting the screen pixel with the highest amplitude value for each instantaneous FFT. The selected pixels were superimposed on the spectrogram, checked visually and corrected if necessary. Time, frequency and amplitude values for each pixel were saved as a csv-file. From the csv-files, several song and syllable parameters were calculated using a custom-written Matlab routine (by SvM).

To get the average frequency of a syllable, we calculated the centre of gravity of all the frequency values (frequency COG). The frequency COG is a weighted average of the frequency, where the relative amplitude of each frequency value is taken into account. The higher the amplitude of a frequency, the stronger this frequency will contribute to the weighted average. It is calculated as
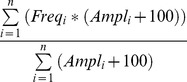
where *n* is the number of frequency and amplitude values in the respective syllable data frame, *Freq_i_* and *Ampl_i_* are the frequency and amplitude values at *i*.

We decided against the use of a more automatic analysis of our recorded vocalisations (provided by software like SASLab Pro, Avisoft Bioacoustics, Germany, or Raven, Cornell Lab of Ornithology, USA), because recordings of ground-dwelling animals (as opposed to flying bats or perching birds) are often too cluttered by background noises resulting from movement of litter, so that not all vocalisations can be correctly detected automatically.

We found a dichotomous distribution of the mean frequency of recorded vocalisations, with a small set of sounds around 20 kHz and the second set starting at about 45 kHz (suppl. Figure S2). Typical USVs of wild mice described in the literature [37], [38] are above 45 kHz. The sounds from around 20 kHz are probably produced by unspecific exhaling. Accordingly, we excluded all vocalisations lower than 45 kHz from further analysis. All remaining syllables showed a normal distribution in mean frequency (One-sample Kolmogorov-Smirnov test, df = 1, p<2.2e-16). This is in contrast to Hoffmann and colleagues [37], who found a distinction into low- and high-frequency syllables, with a cut-off frequency of 90 kHz. As we did not find such a distinction, we did not divide our USV syllables into low-frequency and high-frequency syllables. The difference between the frequency distributions of the study of Hoffmann and colleagues [37] and ours might result from the different sub-species of wild house mice used: In the former study *Mus musculus musculus* was recorded, in our study *Mus musculus domesticus*.

### Statistical analysis

For statistical analysis we used three data sets. The two temporal data sets had one row for each mouse in each recording situation and recording night. The spectral data set had one row for each of the 4,865 syllables. The first temporal data set contained the number of all songs each mouse sang in the three different social contexts and in the two recording nights, including those mice that did not sing at all. The second temporal and the spectral data set only contained those mice, that had emitted at least three songs. For an overview of all parameters of the three data sets see table 1.

To analyse these data sets, we used a two-step approach. In the first step, we tested, if social context, social familiarity or mouse identity had an influence on the temporal and spectral parameters of mouse USV. This was in order to determine, if the amount and type of USV was dependent on the situation, the individual or both. In the second step we tested if population or sex had an influence on mouse USV. For both steps we applied a PERMANOVA (PERmutational Multivariate ANalysis Of VAriance). PERMANOVA is a multivariate, multifactorial analysis of variance for non-parametric data that uses permutations (function ADONIS of the R package VEGAN [39]). We ran the analysis separately for each data set with 5,000 permutations for each run.

For visualisation of the data we ran linear discriminant analyses (function LDA of the R package MASS) and plotted the first two discriminant functions for each data set.

As some of the analysed data were not normally distributed (Shapiro-Wilks p<0.05), we applied non-parametric statistical methods throughout (post-hoc tests: Wilcoxon signed rank test and Wilcoxon rank-sum test). Where necessary, we corrected the obtained p-values for multiple testing, using Bonferroni correction (p′ = p * number of tests). All statistical tests were carried out using R 2.14.1 [40].

### Syntax analysis

To analyse the syntax of the recorded songs we compared the recorded syllable sequences with syllable sequences we generated using two models, a simple Probability model (PM) and a Markov model (MM). We analysed (1) how likely a certain syllable type was used by a group in general, and (2) how likely a sequence of syllables begins or ends with a certain syllable type. We further analysed (3) the number of repetitions of each syllable type and (4) the occurrence of syllable type-doublets and syllable type-triplets. Below, we will explain in detail how we determined the number of syllable types we used and how we generated syllable sequences using the two models PM and MM.

#### Number of syllable types

**Figure 2:**
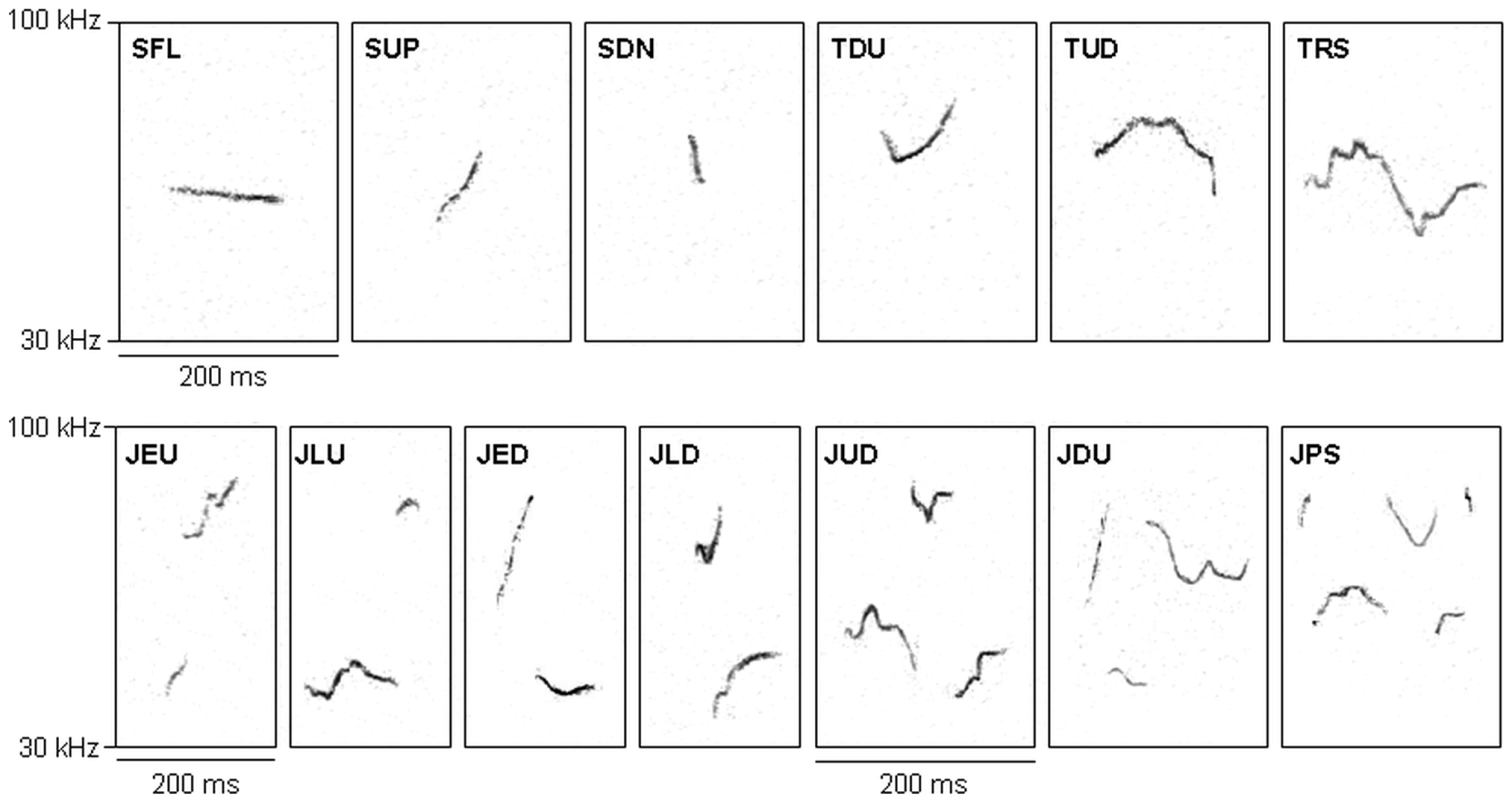
Spectrograms of the 13 syllable types. Spectrograms were generated with 256(FFT) using the software Selena (Department of Animal Physiology, University of Tübingen; Germany). For abbreviations see table 2.

To find a reasonable number of syllable types, we tested a 5-syllable-type model against a 13-syllable type model (Figure 2). We defined 5 or 13 syllable types, respectively, using the extracted parameters and comparable criteria that have been used so far (see e.g. [12], [41]). We assigned each syllable to one of those types for both models. Our types and criteria for the 5-type model were as follows: **Jumps**, syllables that have one or more frequency jumps (at least 20 kHz change in less than 4 instantaneous FFT bins); **Turns**, syllables that have one or more frequency turns (one turn consisting of two frequency changes, each at least 0.8 kHz in less than 3 ms); **Up**, syllables with an upward frequency modulation (at least 0.05 kHz per 1 ms); **Down**, syllables with an downward frequency modulation (at least 0.05 kHz per 1 ms); **Simple**, all other syllable types. As visual inspection of syllables suggests that especially turns and jumps are much more variable, we additionally generated the 13-syllable type model. For this, we counted jumps in the first half of the syllable and jumps in the second half of the syllable, applying the same criteria as above. We also differentiated between jumps going up and jumps going down. This resulted in seven different jump syllable types, depending on whether there was a jump in the first (early jumps), in the second (late jumps) or in both halves and if these jumps were going up or down, or if there were more than two jumps in one syllable. If, for example, a jump to a higher frequency occurred in the first half of the syllable, this syllable was assigned to the **Jump-Early-Up** (JEU) type; if a jump to a higher frequency occurred in the second half of the syllable this would be a **Jump-Late-Up** (JLU) syllable. The same principle applies for syllables with two frequency jumps. A syllable in which the first jump is upwards and the second jump downwards would be a **Jump-Up-Down** (JUD) syllable. To distinguish between different types of turn syllables, we differentiated between syllables with a U-shaped turn, syllables with a turn in the opposite direction and syllables with more than one turn. For the resulting syllable types see figure 2 and table 2.

**Table 2:** Syllable types.

			German females			German males			French females			French males		
5 Syllable Types	13 Syllable Types	Abbr.	p(1)	p_Sta_ (2)	p_Sto_ (2)	p(1)	p_Sta_ (2)	p_Sto_ (2)	p(1)	p_Sta_ (2)	p_Sto_ (2)	p(1)	p_Sta_ (2)	p_Sto_ (2)
Simple	Simple-flat	SFL	0.05	0.10	0.09	0.04	0.02	0.06	0.05	0.09	0.11	0.09	0.12	0.12
Down	Simple-down	SDN	0.03	0.06	0.07	0.05	0.07	0.06	0.03	0.03	0.06	0.11	0.14	0.14
Up	Simple-up	SUP	0.33	0.44	0.47	0.46	0.54	0.57	0.29	0.50	0.49	0.38	0.30	0.35
Turn	Turn-down-up	TDU	0.01	0.01	0.02	0.03	0.03	0.03	0.03	0.05	0.07	0.04	0.06	0.06
	Turn-up-down	TUD	0.11	0.16	0.10	0.15	0.19	0.14	0.09	0.16	0.11	0.20	0.17	0.24
	Turn-multi	TRS	0.16	0.12	0.17	0.12	0.09	0.10	0.06	0.06	0.02	0.08	0.11	0.05
Jump	Jump-early(3)-down	JED	0.10	0.00	0.00	0.09	0.03	0.02	0.15	0.03	0.04	0.02	0.05	0.00
	Jump-late(3)-down	JLD	0.03	0.06	0.01	0.02	0.01	0.00	0.05	0.03	0.03	0.05	0.03	0.00
	Jump-down-up(4)	JDU	0.05	0.02	0.04	0.02	0.00	0.00	0.10	0.00	0.02	0.00	0.00	0.00
	Jump-early-up	JEU	0.00	0.00	0.00	0.00	0.00	0.01	0.01	0.00	0.00	0.02	0.03	0.05
	Jump-late-up	JLU	0.00	0.00	0.00	0.00	0.00	0.00	0.01	0.00	0.00	0.01	0.00	0.00
	Jump-up-down	JUD	0.00	0.00	0.00	0.00	0.00	0.00	0.01	0.00	0.00	0.00	0.00	0.00
	Jump-multi^(5)^	JPS	0.12	0.02	0.02	0.00	0.01	0.00	0.12	0.04	0.04	0.00	0.00	0.00

(1) Overall probability to emit this syllable type;

(2) probability to start or stop a sequence with this syllable type;

(3) early jumps occur in the first half of the syllable, late jumps occur in the second half;

(4) one jump down in the first half of the syllable, one jump up in the second half of the syllable; (5) these syllables have more than two jumps.

Given are names, abbreviations and the probabilities of groups to use, start or stop with these syllable types.

We applied a cluster analysis to validate our predefined syllable types. Syllables were clustered according to their original shape, not their derived acoustic parameters. To compare the frequency-time-courses of syllables we applied a dynamic time-warping method. For this, we stretched all syllables to a standard length of 50 ms, re-interpolating the missing values, and shifted them to the same mean frequency of 80 kHz (custom Matlab routine by SVM). We then used a dynamic time warping algorithm to find the shortest possible distance between all pairs of syllables (R package dtw [42]). Dynamic time warping measures the distance between two time series in a non-linear way, i.e. it stretches or compresses them locally in order to make them as similar as possible. The distance between the two time-warped time series is then computed by summing up the distances of the individual aligned elements [43], [44]. In our case, the time series are the frequency-time-courses of the syllables, and the distance measured is the frequency difference between the syllables.

**Figure 3:**
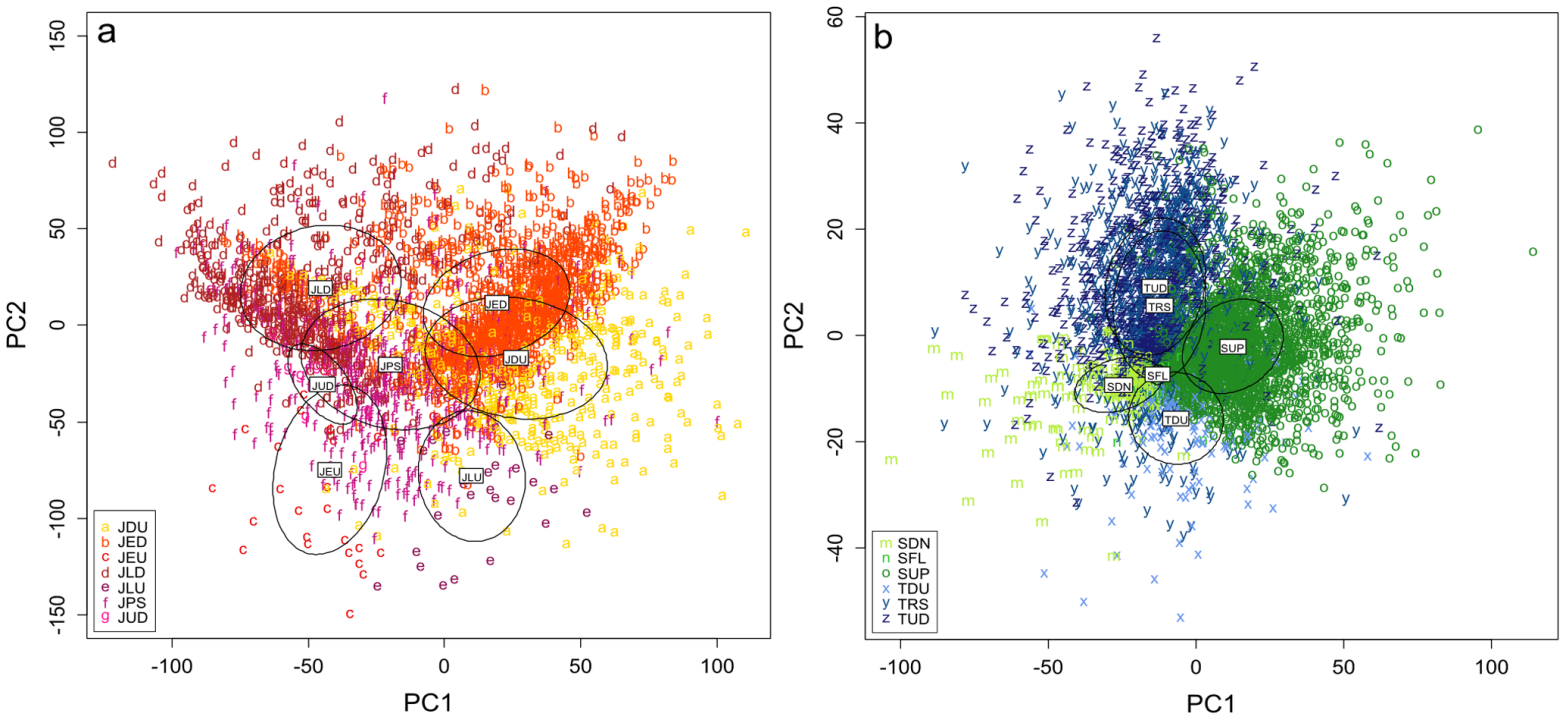
Principal component analysis of syllable types. Scatter plots are based on the spectral parameters of the main analysis (see table 1). **a** Jump syllable types. 1^st^ principal component (PC) distinguishes between early and late jumps, 2^nd^ PC distinguishes between upward and downward jumps. **b** All non-jump syllable types. 1^st^ PC distinguishes between upward and downward frequency modulations, 2^nd^ PC distinguishes between u-shaped and inverse-u-shape frequency modulations. For explanation of abbreviations see table 2.

The cluster analysis of the 5 syllable types showed a decent clustering of syllable types Simple, Down, and Up, but rather dispersed clusters for syllable types Turn and especially Jump. The cluster analysis of the 13 syllable types proved to be significant and also a visual examination of clustering showed a reasonable clustering of syllable types (Figure 3). We thus decided to use these 13 syllable types in the syntax analysis. For this we extracted the syllable sequences from the songs we recorded, separated for the four groups (2 populations, 2 sexes).

#### The Probability model and the Markov model

For the Probability model, we used the overall probabilities of syllable types to occur in the song of the four groups of mice (for syllables types and their abbreviations see table 2 and figure 2). From these we calculated the expected number of syllable type repetitions and the expected occurrence of syllable type-doublets (e.g. SDN>SDN, SDN>TDU or TUD>JPS) and syllable type-triplets (e.g. SDN>SDN>SDN, SDN>TDU>TRS or TUD>JPS>JPS). As an example, the expected probability *p* of occurrence of the triplet SDN>TDU>TRS would be calculated as *p*  =  p_SDN_ * p_TDU_ * p_TRS_.

Another simple model to analyse syllable sequences is the Markov model. Markov models do not use the probabilities of the occurrence of certain syllable types (i.e. the states of a MM), but the probabilities of transitions between different syllable types (states) [45]. From the observed syllable sequences of the four groups, we calculated the probabilities to start and to stop a sequence with a certain syllable type, and the transition probabilities between the 13 syllable types. Using these probabilities, we generated 10,000 sequences for each of the four groups from the model of the respective group (see below for details). From the generated sequences, we calculated the expected syllable type repetitions and expected occurrence of syllable type-doublets and triplets. As an example, the expected probability *p* of occurrence of the triplet SDN>TDU>TRS would be calculated as *p*  =  p_SDN_ * p_SDN>TDU_ * p_TDU >TRS_.

To generate the sequences after the MM, we used the start matrix (containing the probabilities to start a sequence with a certain syllable type), and the transition matrix (containing the probabilities to go from one syllable type to another) and followed this algorithm: to find the first syllable type used, a random number r is uniformly sampled from 0 to 1. If *r<p_Start_S1* (where S1 is the first syllable type in our sorting and *p_Start_S1* the probability to start a sequence with it), S1 is selected as starting syllable; if *p_Start_S1<r<p_Start_S1+p_Start_S2*, S2 is selected as starting syllable; this process is continued until the second last possible case where 
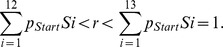
In this case S13 is selected as starting syllable. All following syllables can be selected similarly according to the transition probabilities between the 13 syllable types. As each syllable can act as a stop syllable, the transition probabilities from one syllable type to any of the 13 syllable types do not add up to 1. Therefore a case can occur where
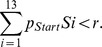
In this case, the syllable sequence ends with the last syllable that has been selected. We set the maximum number of syllables in one sequence to 20, as no syllable sequence we recorded was longer than 19 syllables. So, when the generated sequence reaches the number of 20 syllables, the algorithm will terminate the sequence, no matter which is the last syllable type.

## Results

The ultrasound recording approach applied in our experiments (see Methods section *Recording schedule*) differed from that of previous studies in two major aspects to create more natural situations. First, we recorded always from both animals in the respective social context and started recording only after these animals had two days to get acquainted with the recording environment. Second, we use much longer recording times (two consecutive nights) to assess whether the familiarity gained in the first night had an influence on USV in the second night. The three social context situations focussed on the following main questions. (1) The different sex - same population (DiffSex) context reflects a normal mate encounter situation. Here we can study specific songs emitted by both sexes in a mate choice situation. (2) The different sex - different population (DiffPop) context would not occur naturally since the populations live in allopatry, but allows to judge in comparison with the DiffSex context whether possibly alien USVs provide the same mating stimulus or not. (3) The third context (same sex - same population, SameSex) was designed to record the USV patterns in a social communication context and to compare these between the populations and the mate encounter situation.

### Temporal analysis

We find an overall strong correlation between the number of songs individual mice emitted in the three different social contexts (Pearson's correlation coefficient: DiffPop vs. DiffSex: 0.53; DiffPop vs. SameSex: 0.60; DiffSex vs. SameSex: 0.28). Two of the 27 mice that were used for the final analysis did not sing in any social context or recording night (a French and a German male, Table 3).

**Table 3:** Number of songs emitted by each individual, summed over (Sum) and separated by social contexts.

group	individual^(1)^	Sum	DiffPop(2)	DiffSex	SameSex
German females	CB304F1b3	25	3	22	0
	CB302F1b4	40	24	11	5
	CB309F1c3	86	16	45	25
	CB306F1a1	167	50	60	57
	CB301F1b1	186	45	70	71
	CB308F1b2	225	60	68	97
German males	CB305F1b1	0	0	0	0
	CB300F1b3	27	13	13	1
	CB309F1c2	75	41	33	1
	CB302F1a2	90	19	58	13
	CB303F1a1	115	36	59	20
	CB301F1a2	115	45	52	18
French females	MC507F1a1	22	7	9	6
	MC514F1b8	66	13	22	31
	MC508F1a1	73	35	27	11
	MC505F1b3	81	23	19	39
	MC504F1c1	113	45	50	18
	MC513F1a1	140	26	89	25
	MC500F1a1	195	56	30	109
French males	MC514F1b7	0	0	0	0
	MC503F1c1	3	2	0	1
	MC509F1a1	8	0	5	3
	MC501F1b2	12	1	1	10
	MC412F1c1	30	9	15	6
	MC512F1b4	45	23	7	15
	MC504F1a3	107	6	94	7
	MC509F1b2	495	97	337	61

1) Within groups, individuals are ordered according to increasing number of songs emitted.

(2) DiffPop  =  Different population, different sex; DiffSex  =  Same population, different sex; SameSex  =  Same population, same sex.

Mouse identity had a significant influence on the number of songs emitted (PERMANOVA: F(23) = 14.309, p<0.001). Some individuals (mainly females of both populations) showed no overlap between each other in their values of this and other temporal parameters (mainly song duration), independent of social context.

Social context showed a trend to have an influence on the number of songs emitted (PERMANOVA: F(2) = 2.314, p = 0.078). Mice emitted more songs in the DiffSex situation than in the other two situations. To find the factors responsible for these differences, we conducted post-hoc tests (Wilcoxon signed rank test). After correcting for multiple testing (Bonferroni), this difference was only significant in the DiffSex vs. SameSex comparison (V = 784.5, p′ = 0.023) and trend for the other two comparisons. Social familiarity (comparison of the two recording nights) had no influence on the number of songs emitted (F(1) = 2.324, p = 0. 612). In previous studies, an influence of familiarity on the number of songs was found. The direction of this influence (i.e. more or less songs in familiar situation) differed between studies and sexes. We thus additionally conducted an analysis separated by sex. Neither for females nor males we found an influence of familiarity on the number of songs. We thus conducted the following analysis separated by social context but not by familiarity.

**Figure 4:**
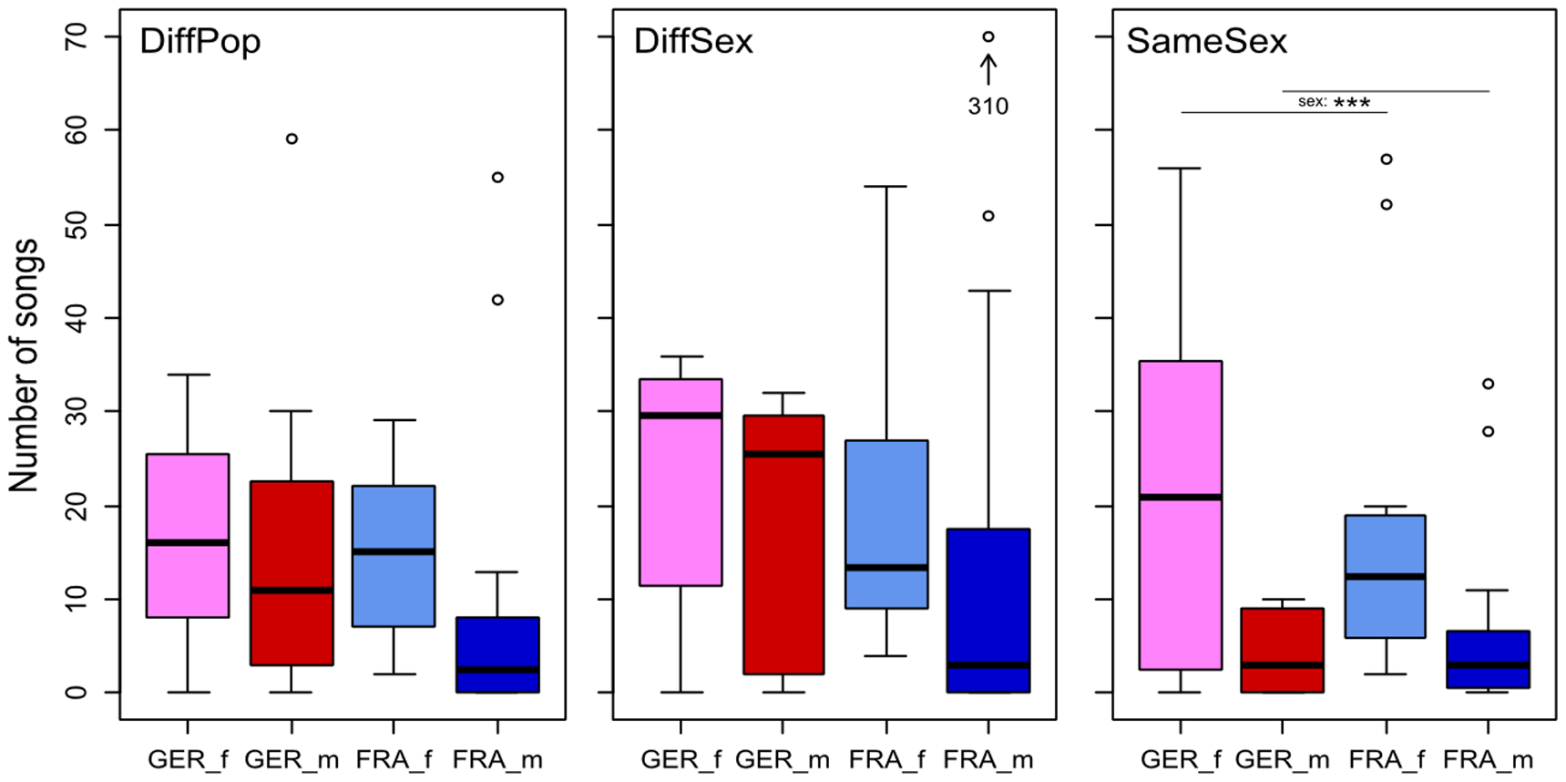
Number of songs emitted in the different social contexts. Box plots are separated by sex and population. DiffPop = Different population, different sex; DiffSex = Same population, different sex; SameSex = Same population, same sex. GER  =  German mice (f = females in pink, m = males in red), FRA = French mice (females in light blue, males in blue). Asterisks denote the cases where found differences were significant (p≤0.001 (***)). A tentative removal of the outlier mouse (310 songs in DS) did not change the results of the statistical analysis.

German mice tended to emit more songs than French mice (Figure 4). This effect was, however, not significant in any of the social contexts (PERMANOVA: all p>0.1). In all situations, females emitted more songs than males of the same population (Figure 4). This influence of sex on the number of songs was only significant in the SameSex situation (PERMANOVA: SameSex, sex: F(1) = 13.021, p = 0.001; all other p>0.1).

**Figure 5:**
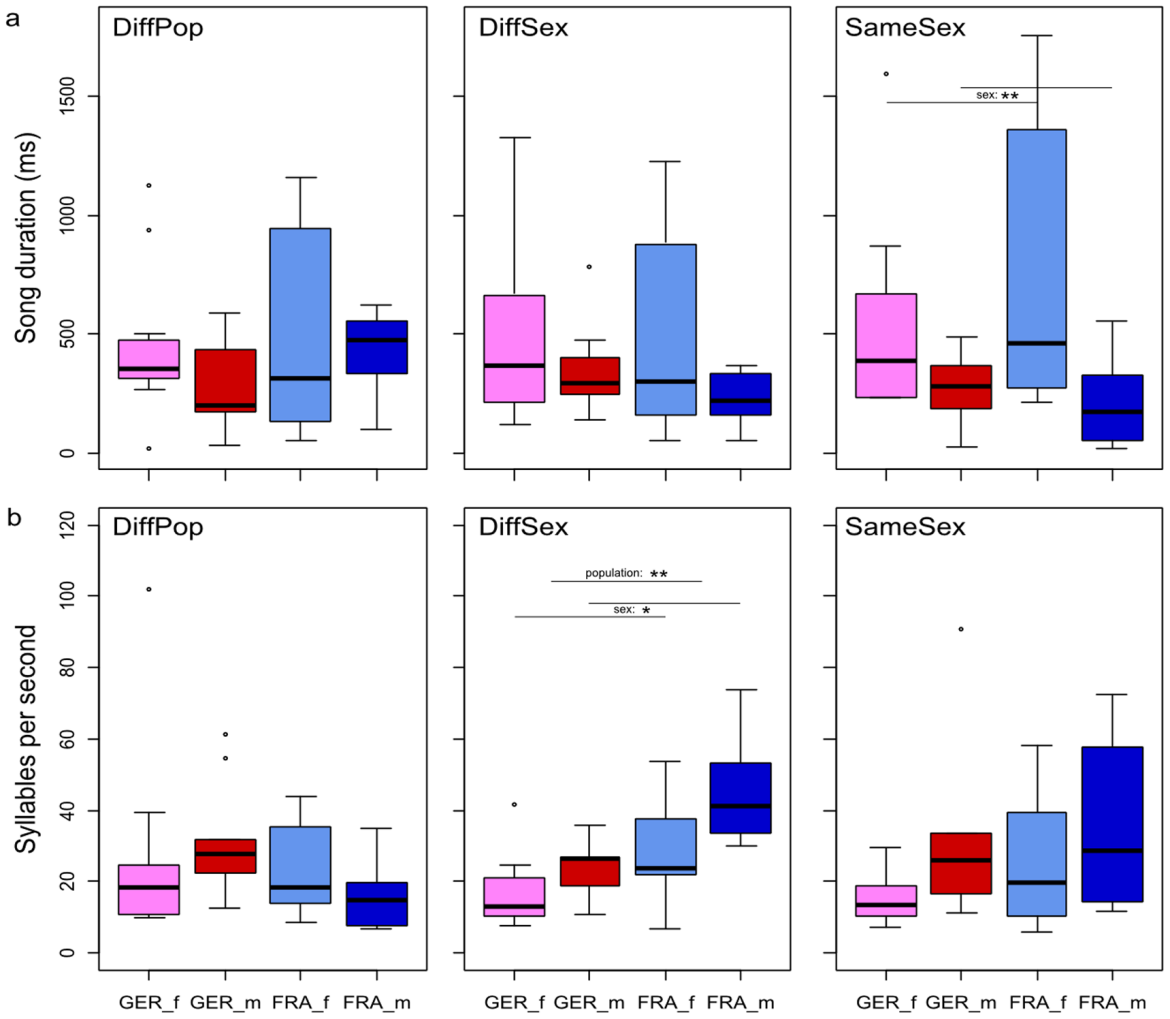
Song duration (a) and syllables rate (b) in the different social contexts. Box plots are separated by sex and population. Abbreviations and colours as in Figure 4. Asterisks denote the cases where found differences were significant (p≤0.05 (*), p≤0.01 (**)).

The other two temporal parameters (song duration and syllables per second) were analysed separately of the number of songs, as only those mice that had emitted at least three songs in the respective recording night, were included into the analysis. Neither social context nor social familiarity had an influence on song duration or syllables per second (PERMANOVA: all p>0.1; Figure 5).

In the DiffSex situation there was a significant influence of both population and sex on temporal parameters (PERMANOVA: population: F(1) = 6.007, p = 0.010; sex: F(1) = 5.700, p = 0.011; Figure 5). In the SameSex situation only the influence of sex was significant (PERMANOVA: sex: F(1) = 6.073, p = 0.008; all other p>0.1). In the DiffPop situation, neither population nor sex had an influence on any these two temporal parameters.

To find the factors responsible for these differences, we conducted post-hoc tests (Wilcoxon rank-sum test) for the DiffSex and the SameSex situations and corrected for multiple testing (Bonferroni, DiffSex: p′ = p*4, SameSex: p′ = p*2). In the SameSex situation females emitted longer songs than males (W = 245, p′ = 0.0085). In the DiffSex situation males emitted more syllables per second than females of the same population (W = 105.5, p′ = 0.0480) and French mice more syllables per second than German mice (W = 98, p′ = 0.0154).

### Spectral analysis

Mouse identity had a significant influence on the syllable parameters (PERMANOVA: F(23) = 65.836, p = 0.0002). The parameters having the biggest influence on separating the data were slope, number of turns and number of jumps. Also the social context had a significant influence on the syllable parameters (PERMANOVA: F(2) = 7.882, p = 0.0002). There was a significant difference between all three combinations of social contexts (DiffPop - DiffSex: F(1) = 6.952, p = 0.0018; DiffPop - SameSex: F(1) = 4.850, p = 0.0102; DiffSex - SameSex: F(1) = 11.757, p = 0.0006). Social familiarity (recording night) had no influence on the syllable parameters (F(1) = 1.734, p = 0.149). We thus conducted the following analysis separated by social context but not by familiarity.

In all three social contexts both population and sex had a significant influence on the syllable parameters (for the results of the model see table 4). In the DiffPop and the SameSex situation there was also a significant but small interaction between population and sex. As can be seen from the F- and R^2^-values of the models, sex was the factor that separated the data best in all three social contexts.

**Table 4:** Model result of PERMANOVA for spectral syllable parameters.

Social context	factors	F (df = 1)	R2	p
DiffPop(1)	pop	7.645	0.004	***0.0004***
	sex	77.183	0.045	***0.0002***
	pop:sex	13.860	0.008	***0.0002***
DiffSex	pop	30.754	0.017	***0.0002***
	sex	73.443	0.039	***0.0002***
	pop:sex	1.177	0.001	0.2879
SameSex	pop	22.907	0.014	***0.0002***
	sex	113.283	0.070	***0.0002***
	pop:sex	10.429	0.006	***0.0002***

(1) DiffPop  =  Different population, different sex; DiffSex  =  Same population, different sex; SameSex  =  Same population, same sex.

Significant p-values in bold-italics (for abbreviations of social contexts see Table 3).

**Figure 6:**
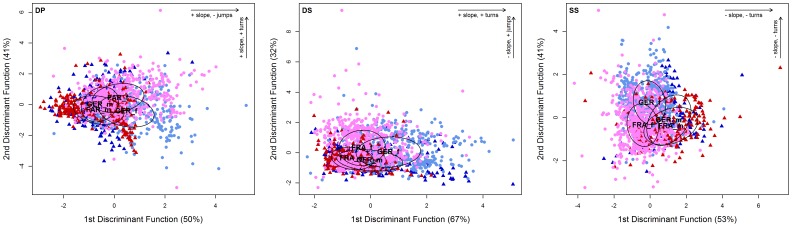
Discriminant function analysis of syllable parameters in the different social contexts. Scatter plots are separated by sex and population. Arrows indicate the direction of the three parameters with the strongest influence on the separation of the data: slope, jumps, turns; plus and minus indicate a positive or negative change of the parameter in the arrow's direction; for the loadings of these and the other parameters see table 5. Abbreviations and colours as in Figure 4.

The LDA revealed that the main factors influencing the difference between females and males were the frequency slope, the number of jumps and the number of turns (Table 5, Figure 6).

**Table 5:** Analysis of qualitative parameters, separated by social context.

		Mean of groups				Loadings of functions			Population difference(3)			Sex difference(3)		
Context	Parameters	GER_f(1)	GER_m	FRA_f	FRA_m	LD1	LD2	LD3	W(4)	p' (*8)	sig.	W	p' (*8)	sig.
DiffPop(1)	duration	72.022	46.050	53.383	47.531	−0.009	−0.032	−0.003	361127	0.0014	**	262951	0.3180	n.s.
	starting frequency	77.422	80.358	78.186	82.657	0.080	−0.020	0.012	344731	0.3646	n.s.	257697	0.0625	n.s.
	frequency slope(2)	0.022	0.196	0.179	−0.004	0.159	1.078	-1.217	278099	0.0000	***	314160	0.0011	**
	minimum frequency	66.513	75.416	67.197	74.674	−0.152	−0.036	−0.051	374026	0.0000	***	260947	0.1773	n.s.
	frequency band	22.500	14.810	23.666	16.180	−0.087	0.007	−0.014	301360	0.0698	n.s.	295967	0.6646	n.s.
	frequency COG(3)	75.011	83.405	77.913	83.381	0.117	0.042	0.049	352005	0.0442	trend	264501	0.4819	n.s.
	number of jumps	0.629	0.125	0.734	0.251	−0.532	0.332	1.115	302061	0.0177	*	287583	2.8360	n.s.
	number of turns	2.069	1.369	2.017	1.084	0.025	0.458	−0.513	334248	2.9200	n.s.	260500	0.1306	n.s.
		Proportion of trace:				0.5152	0.4061	0.0787						
DiffSex	duration	69.299	45.574	46.020	29.772	−0.035	−0.010	−0.011	495472	0.0000	***	423758	0.0000	***
	starting frequency	79.184	78.511	76.044	75.979	0.007	−0.078	−0.078	469865	0.0000	***	313569	1.9128	n.s.
	frequency slope(2)	0.075	0.238	0.164	0.325	0.396	−0.918	−0.383	339871	0.0001	***	274447	0.0000	***
	minimum frequency	69.409	73.667	66.275	71.855	−0.117	0.088	0.036	458440	0.0000	***	251426	0.0000	***
	frequency band	23.252	15.489	22.904	15.529	−0.044	0.075	0.110	371915	1.0400	n.s.	432694	0.0000	***
	frequency COG(3)	79.294	81.399	76.898	80.425	0.094	−0.038	0.029	449380	0.0000	***	265302	0.0000	***
	number of jumps	0.553	0.144	0.712	0.135	0.178	0.798	−1.311	348256	0.0000	***	426870	0.0000	***
	number of turns	2.011	1.069	1.669	0.782	0.204	0.065	0.280	419100	0.0212	*	417427	0.0000	***
		Proportion of trace:				0.658	0.333	0.009						
SameSex	duration	67.089	48.974	52.574	28.722	−0.002	0.031	0.029	294371	0.0020	**	206936	0.0902	trend
	starting frequency	78.299	83.913	74.850	86.112	0.028	−0.113	0.085	241756	0.0358	trend	240086	0.4559	n.s.
	frequency slope(2)	0.091	−0.072	0.083	0.012	−0.973	−0.833	0.092	282895	0.1986	n.s.	208738	0.1749	n.s.
	minimum frequency	69.478	78.026	62.571	80.645	−0.020	0.190	−0.128	321772	0.0000	***	199929	0.0041	**
	frequency band	23.054	10.809	22.291	12.899	−0.041	0.108	−0.119	248630	0.3710	n.s.	232757	2.8520	n.s.
	frequency COG(3)	79.509	84.113	72.288	87.663	0.061	−0.065	0.017	312620	0.0000	***	209621	0.2371	n.s.
	number of jumps	0.605	0.061	0.821	0.118	−0.120	0.096	−0.380	222315	0.0000	***	239387	0.2715	n.s.
	number of turns	1.992	1.200	2.236	0.763	−0.059	−0.371	−0.039	244939	0.0959	trend	243429	0.1270	n.s.
		Proportion of trace:				0.5366	0.3964	0.0671						

(1) DiffPop  =  Different population, different sex; DiffSex  =  Same population, different sex; SameSex  =  Same population, same sex. FRA  =  French, GER  =  German, f  =  female, m  =  male.

(2) Calculated as change of frequency over time (kHz/ms).

(3) Centre of gravity, see Methods section *Sound analysis* for calculation.

(4) Post-hoc Wilcoxon rank-sum tests; corrected p-values.

To find the parameters that are significantly different between the sexes or populations, we conducted post-hoc tests (Wilcoxon signed rank test with Bonferroni correction for multiple testing). Mice from the German population emitted consistently longer syllables than mice from the French population, this was significant in all three social contexts (for the statistics of all post-hoc tests see Table 5). German mice also used more syllables with turns than French mice, this was only significant in DiffSex and trend in SameSex. French mice used more syllables with jumps than German mice, this was significant in all three social contexts. For the slope parameter, there was an interaction of population and social context: slope was more positive in German mice than French mice in the DiffPop, but more positive in French than German in DiffSex.

Female mice emitted consistently longer and more structured syllables, i.e. syllables with a wider frequency band, more turns and more jumps than male mice in all three social contexts. Male mice emitted consistently syllables with a higher minimum frequency than female mice, this was significant in DiffSex and SameSex. Again, there was an interaction for the slope parameter: slope was more positive in male mice than female mice in DiffSex, but more positive in female mice than male mice in SameSex, it was equal in DiffPop.

### Syntax analysis

**Figure 7:**
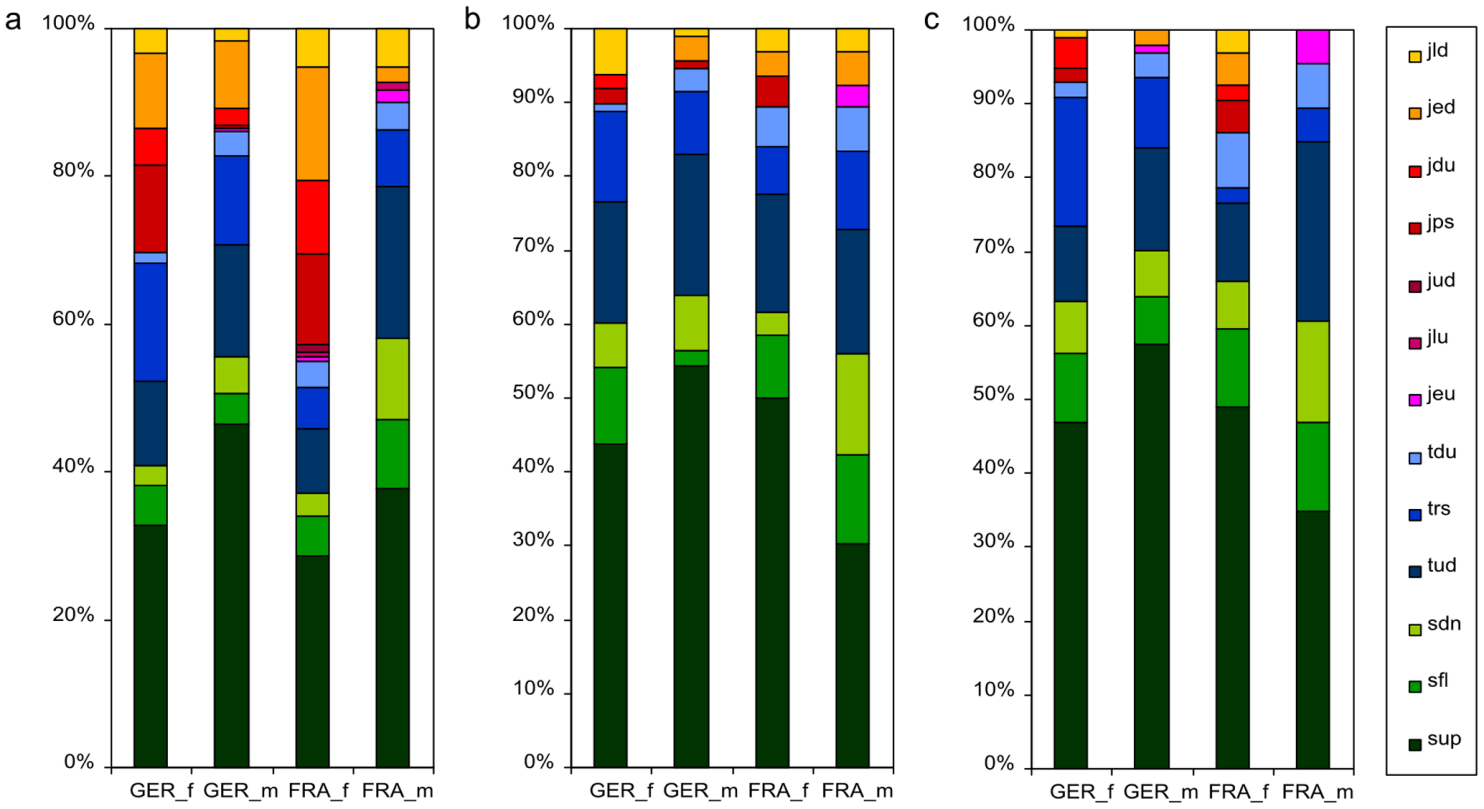
Probabilities to (a) use, (b) start with or (c) end with a certain syllable type. Separate bars for each group (FRA = French, GER = German, f = female, m = male). Different colours for different syllable types: greenish colours for simple syllable types, bluish colours for turn syllable types, reddish-yellowish colours for jump syllables. For syllable type abbreviations see table 2.

We compared the general probability to use certain syllable types, and the probabilities to start and to stop a sequence with certain syllable types (Figure 7). The general probability to use certain syllable types was different between populations and between sexes (Chi-squared test: population χ^2^(12) = 58.721, p′<0.001; sex: χ^2^(12) = 132.863, p′<0.001; p-values corrected for multiple testing). The probabilities to start a sequence with certain syllable types was not significantly different between populations and sexes (populations χ^2^(10) = 12.283, p′ = 1.600; sexes: χ^2^(10) 15.696, p′ = 0.652). The probabilities to stop a sequence with certain syllable types was borderline significantly different between populations and sexes (populations χ^2^(10) = 24.051, p′ = 0.045; sexes: χ^2^(10) = 23.669, p′ = 0.051). Generally, German mice used more turn syllables and French mice more jump syllables. Females used more jump syllables and males more upward modulated syllables and turn syllables. The start and stop syllables were quite similarly distributed like the general usage. However, both populations and sexes used less jump syllables to start or stop a song than would have been expected from the general usage of these syllable types.

**Figure 8:**
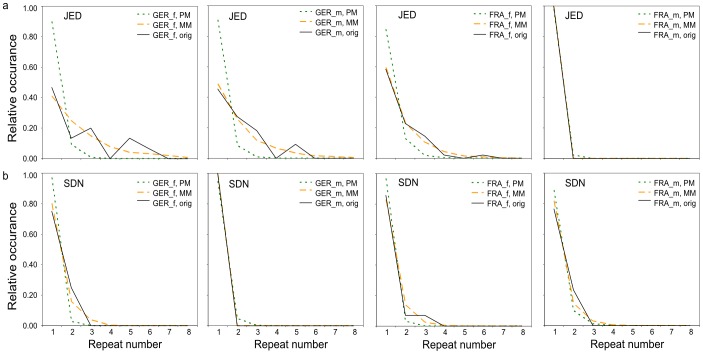
Comparison of the repeat number distribution of syllable types. Presented are graphs for the syllable types (a) JED (Jump-early-down, for details see Table 2) and (b) SDN (Simple-down), with separate graphs for each group (FRA = French, GER = German, f = female, m = male). The solid black lines show the distribution of repeat numbers in the observed syllable sequences (orig). The dotted green and dashed yellow lines show the distribution of repeat numbers in the syllable sequences calculated with the Probability model (PM) and with the Markov model (MM) respectively.

We further analysed the distribution of syllable repeats. We compared this measure against the syllable sequences generated by the two models (PM and MM). The syllable-sequences generated by the two models gave in some cases a rather good representation of the real repetition rates (Figure 8). In general, MM gave a better fit than PM. The distance between the real data and the models was d_SUM-PM_  =  9.787 for the PM and d_SUM-MM_  =  4.278 for the MM, calculated as the absolute differences between the occurrence of repeat numbers in a model and the occurrence of repeat numbers in the original, summed over repeat numbers and syllable types, i.e. e.g. d_SUM-PM_ *= ∑∑|PM(repeatnumber) - original(repeatnumber)|*. Both models were inaccurate in cases where a higher repetition rate was more likely than a lower one (e.g. five repetitions of JED were more common than four repetitions of JED in German mice of both sexes, see figure 8). If calculated separated by groups, the results were fairly similar for each of the groups.

**Figure 9:**
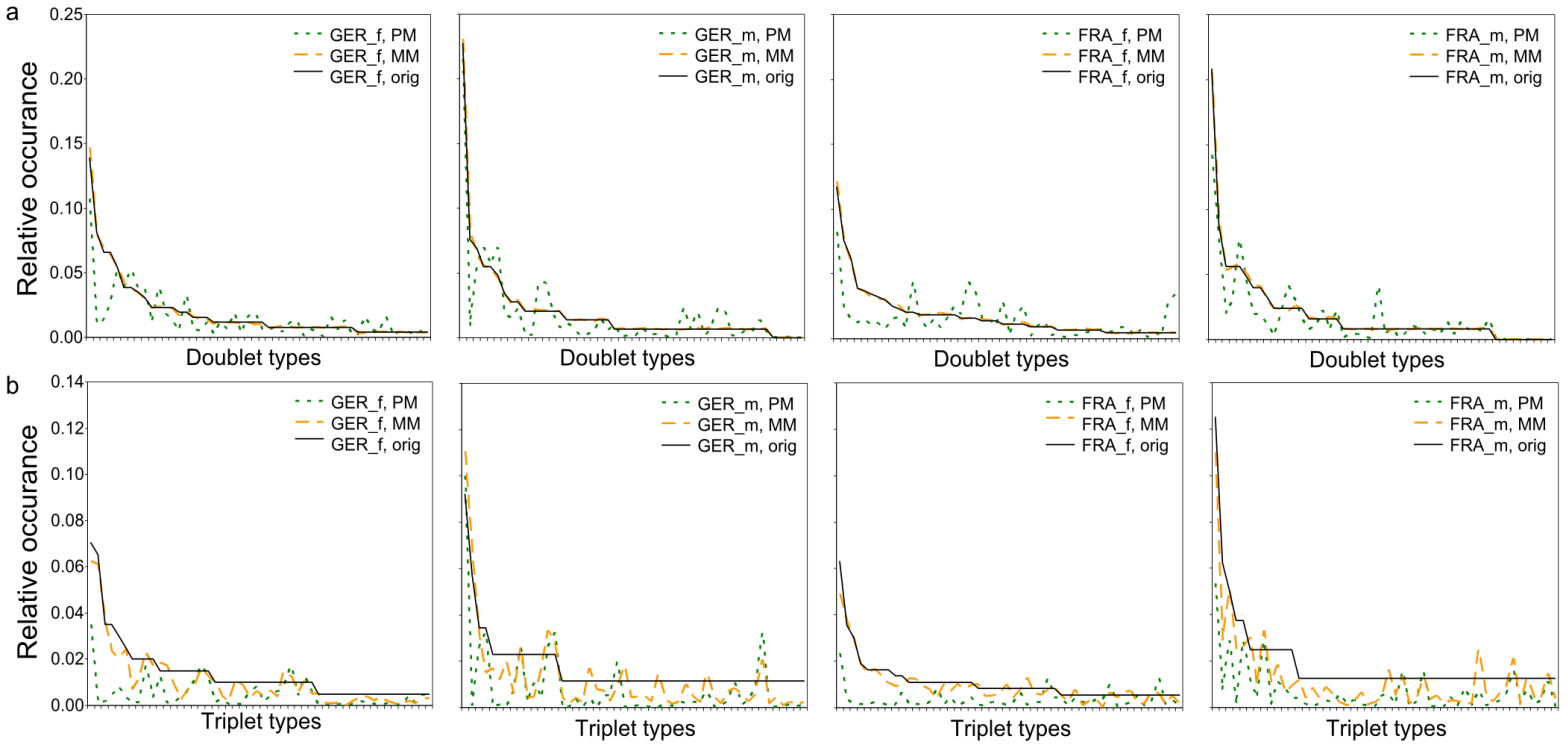
Distribution of doublets (a) and triplets (b) of syllable types. Doublets and triplets are sorted according to decreasing probability in the real data. Separate graphs for each group (FRA = French, GER = German, f = female, m = male). The solid black lines show the distribution doublets and triplets in the observed syllable sequences (orig). The dotted green and dashed yellow lines show the distribution of the respective doublets and triplets in the syllable sequences calculated with the Probability model (PM) and the Markov model (MM) respectively.

The last property of syllable sequences we analysed was the occurrence of syllable type-doublets and syllable type-triplets. We compared this measure against the syllable sequences generated by the two models (PM and MM). The syllable-sequences generated by PM gave a poor representation of the real occurrence of doublets and triplets (Figure 9). The distance between real data and the PM was d_DUPL-PM_ = 0.514 for doublets and d_TRIP-PM_ = 1.027 for triplets, calculated as the absolute differences between the occurrence of doublets (triplets) in the model and the occurrence of doublets (triplets) in the original data, summed over doublet (triplet) types, i.e. e.g. d_DUPL-PM_ *= ∑|PM(doubletoccurance) - original(doubletoccurance)|*. MM was rather accurate for the doublets, but not for the triplets (Figure 9, d_DUPL-MM_ = 0.023, d_TRIP-MM_ = 0.568). In syllable type-doublets only one transition occurs; thus MM is very accurate, as the transition probabilities are the core of a first-order MM, i.e. a MM where one state (syllable type) only depends on its directly preceding state (syllable type). In triplets, however, the pre-preceding state will be important as well, a feature not incorporated in our first-order MM. If calculated separated by groups, the results were fairly similar for each of the groups.

## Discussion

The recorded USV patterns show that the two populations have indeed diverged with respect to the use of ultrasonic songs, akin to the divergence of dialects. In addition, our comparisons of different social context situations provide further interesting insights into the use and potential role of USVs. We will discuss these different points in detail in the following.

### Social context but not familiarity had an influence on number of emitted USV

Some of the mice did not sing at all during the recording sessions. This supports and expands an earlier study, where it has been shown that some mice do not emit USV [38]. In this study, however, mice had been recorded for only 90 minutes, while we can now show that non-singers seem to stay non-singers, even when recorded for extended times. At the same time, we found a strong correlation between the number of songs individual mice emitted in the three different social contexts, recorded at different times. This is an aspect that had not been tested before and it suggests that the propensity to sing is a personal characteristic of an individual. It will be interesting to test in the future whether this propensity changes over the life time of an individual and whether it relates to fitness parameters. In birds, song length but not song rate has been linked to personality [46].

The social context had in tendency an influence on the number of songs the German mice emitted. In the situation where mice were confronted with mice of the same population but different sex (DiffSex), they emitted more songs than in the other two situations (Figure 4). The fact that the different sex context with the respective foreign population elicits fewer songs suggests that the own population cues are indeed more attractive. However, the French mice did not show the same tendency.

Two previous studies showed an influence of several other social parameters on USV production in same-sex encounters of female mice. The number of USV emitted by a female during interaction with an unknown female partner was lower when the mouse was sexually receptive, pregnant or aged [47]. Also the feeding status of both partners had an influence on the number of USV emitted [48]. Generally, female mice produced more USV towards conspecifics that had been fed, than to those that had not. Further, well-fed mice did emit more USV towards conspecifics that had just eaten palatable food, than to those that had just eaten non-palatable food. Food-deprived mice, in contrast, did not make such a distinction. Our results expand these findings by the suggestion that also the sex and population affiliation of the encountered conspecific can influence the amount of USV uttered. Together these studies suggest the need for more experiments addressing the possible information context of USV interactions.

Interestingly, we found no influence of social familiarity on any of the temporal or structural parameters tested. While we know of no study that analysed the influence of familiarity on the structure of USV, some previous studies did find an influence of familiarity on a temporal parameter. Hoffmann and colleagues [49] found that male mice emit more USVs when presented with the urine of unfamiliar than when presented with the urine of familiar females. A study on male-female dyadic encounters had opposing results: here, more USV were uttered on the second of two consecutive encounters [50], i.e. when animals were familiar with each other. In contrast, in a study on female-female dyadic encounters less USV were uttered during the familiar situation of a second encounter with the same individual [51]. However, the latter two studies were conducted with strains of inbred mice, i.e. the results are not directly comparable to ours.

Another major difference between all three aforementioned studies and ours is that the mice in our study were in close contact with each other for two days and nights without interruption. In contrast, the presentation/encounter situations in the other studies lasted only short time frames (between 3 and 30 minutes in the respective studies), and animals were separated between the repeated encounters. This separation could possibly explain the increased/decreased amount of USV on the re-encounter.

Taken together, the results of the previous and our study suggest that on fine temporal scales, differences in the amount of USV uttered are likely. It appears, however, that extended exposure to other mice does not change the general behaviour of an individual, much in line with the assumption that this is more dependent on personality, as discussed above. This is an important conclusion, since it validates our approach to reuse the same animals in different contexts. This result gives also support to the findings of Hoffmann et al. [38] who suggested that USV can signal individuality in wild mice. It would be interesting to analyse, if also structural parameters differ, when measured on a finer temporal scale. The rather small amount of USV the mice in our study uttered in short time intervals, did not allow such a fine scale analysis.

A second conclusion of our finding that social familiarity had no influence on any of the temporal or structural parameters pertains specifically to the same sex contexts. In these situations, one could have expected that the songs serve to establish hierarchy relationships. However, if this were the case, one could have expected that hierarchy is established in the first night, i.e. the second recording night should be different. Since this is not the case, it appears that the songs in the same sex situation serve a more general social communication purpose (see e.g. [48]) that needs to be further explored.

### French and German mice differ in quantity and quality of emitted syllables

The number of songs emitted per night did not differ much between populations in any of the three social contexts. The number of syllables emitted per second on the other hand, differed between populations in the same-population different-sex context, where French mice had higher syllable rates than German mice. Since syllable number was otherwise not population-specific, this appears to point to a special song pattern in the mating context.

In contrast to syllable rates, structural parameters of syllables differed between populations in all three social contexts. German mice emitted longer syllables and more syllables with turns than French mice. French mice, on the other hand, emitted more syllables per second and used more syllables with jumps. In other words, French mice sang faster than German mice (see below for a discussion about syllable rate trade-offs.)

The steepness of the slope of syllables showed a more complex pattern of variation between the two populations in the three different social contexts. In the same-population different-sex situation the slope was significantly more positive in males than in females of both populations. In the same-sex situation it was slightly, but not significantly more positive in female mice of both populations. In the different-population different-sex situation, there was an interaction between population and sex: While in German mice males had a more positive slope, in French mice females had a more positive slope. We cannot rule out that French and German mice influenced each other in the different-population context. It has been shown that male mice housed socially adjust their USV frequency to cage mates [13]. In that study, however, mice had been housed together for several weeks, whereas in our study they were neighbours for only two days. The steepness of the slope has previously been found to differ according to the context [7]. Taken together, these results suggest, that slope is an important contextual parameter in mouse USV.

### Female and male mice emit USV in several social contexts

Our finding that during the same-sex dyadic encounters both sexes emitted calls, is in line with previous studies suggesting that that USV does not only serve as courtship signals to mice [47], [48], [51]. In the same-sex encounters in our study, female mice emitted more songs than males. This finding on wild mice supports the results of two other experiments that analysed the USV in dyadic encounters of inbred mouse strains [7], [52], who also found the highest amount of USV in the female-female dyadic encounters, as opposed to the female-male and male-male encounters. The Gourbal et al. [52] study found in addition that female mice that were separated by a partition (“perforated and transparent partition allowing olfactory and auditory contacts”), showed significantly less USV. As our recordings were always performed with a partition, it could be that we even underestimate the true amount of USV in female-female encounters.

The different amount of USV in same-sex interactions between females and males could arise from differences in their social behaviour. For female mice it is more common to live among other females than for male mice, for example in communal nests [53], [54]. To encounter another female, even an unknown one, could thus be a more common social situation. For a male, on the other hand, another male is more likely taken as an intruder. So far USV in mice has been mainly found during nonaggressive interactions [47], [48], [55]. It thus fits, that male mice might emit less USV in a potentially averse situation. This hypothesis is further supported by results from the above mentioned study by Gourbal et al. [52]: In male-male encounters, they observed only one type of USV syllables (“V”-shaped), that were always shortly followed by sonic calls and fighting. These result can, however, not be compared directly to ours, as we recorded with a partition, hence there was no chance for full physical interaction.

Different-sex dyadic encounters, mainly courtship and mating, are the best studied situations where USV are emitted [5], [6], [55]. Many of these studies, however, analysed only the behaviour of single mice, not dyads. The main focus was also mostly on the song of male mice and the behaviour of female mice, using urine samples to mimic females and song playbacks to mimic males. Those studies that analysed the actual mating behaviour suggest that the USV of male mice helps to avoid the withdrawal of the female before and during copulation and thus facilitate mating [4], [10].

In dyadic encounters, it is difficult to tell with certainty which of the two mice was emitting the recorded calls. One solution is to anaesthetize one individual [7], which however might change the behaviour of the vivid mouse. In our experimental design the interacting mice had no full physical contact, but were still able to smell, hear and to a certain extent also touch the other individual through the grid. In this way we could tell apart which mouse emitted which calls. We could thus disentangle the true amount and structure of USV syllables that female and male mice are emitting in different social situations. To our knowledge, ours is also the first study to include structural parameters in the analysis of same-sex dyadic encounters.

We found that in both of our different-sex dyadic encounter situations (DiffSex, DiffPop), both females and males did emit USV. Further, the amount of USV was similar in males and females. We argue that the production of USV in the mating context is not a unilateral activity directed from the male to the female. It is more likely an interactive process between the two sexes, where the song of both is equally important to stimulate the mating process.

The other temporal and some of the structural parameters were affected by sex as well. Generally, females had longer songs (significant in the SameSex context) with a lower syllable rate, and males shorter songs with a higher syllable rate (significant in the DiffSex context). In other words, females sang slower and males faster.

Female mice of both populations also emitted longer syllables that were more structured than those of males, i.e. syllables had a wider frequency band, more turns and more jumps. Male mice, however, called at higher minimum frequencies than females. The finding that the syllables of female mice are more structured fits to their slower song rate, since animals are usually faced with a trade-off between frequency bandwidth and call rate. The higher the bandwidth (more structured syllables), the less calls can be emitted per interval of time. This has been shown in several animal groups (e.g. song birds [56], bats [57] and mice [58]). It can be largely explained by constraints on vocal production that impose a trade-off between call rate and frequency bandwidth. However, in birds and mice it has been shown that females have a preference for the maximization of both song parameters in males. This can act as selection pressure on male vocalisation [58], [59]. It has still to be tested, if and what general preferences female mice have concerning the structural properties of male song.

In contrast to our results, Hammerschmidt et al. [7], did not find strong differences in the structure of USV between males and females, but they used inbred strains in their analysis and recorded only during the light phase, which is the resting time for mice. This could either suggest that wild mice have a higher repertoire of songs or that the response of the animals during their rest phase is different. In another species of muroid rodents, *Peromyscus californicus*, a difference in frequency and variability of USV between wild and inbred individuals has already been shown [60].

Disregarding possible differences between males and females, it is noteworthy that females appear to be no less active in emitting songs in all contexts. Given that most USV tests are still focussed on males (but see [47], [48], [51]), this observation should lead to a reconsideration of the role of USV in female communication.

Independent of population and sex, we also found differences in the temporal and structural patterns of USV between individuals. These differences proved to be stronger than the differences between different social contexts. In other words, individual mice seem to emit their own typical songs, which are only to a certain extent influenced by the social context. These results give support to the hypothesis that the structure of USV is an individual characteristic of house mice [38]. Further studies are needed to analyse the persistence of these individual differences and their potential relevance for individual fitness.

### Wild mice sing with a complex syntax

A visual inspection of mouse USV hints already at a complex temporal sequencing of syllables. To analyse the statistical properties of these sequences, we compared syllable repetition rates and the occurrence of syllable type-doublets and triplets of the recorded syllable sequences with those of sequences generated by two models, a simple Probability model and a first-order Markov model.

The repetition rates of syllable types were in some cases quite well represented by both models, with the MM always outperforming the PM. The models were, however, inaccurate in cases where a higher repetition rate was more likely than a lower one. This is a feature of the functions underlying these models: The sequences generated by both models result in decreasing functions of the repeat numbers of syllable types (PM: *P_n_ = p^n^*; MM: *P_n_ = r^n-1^(1-r)*; where *P_n_* is the probability of *n* repetitions, *p* the constant giving the probability to emit a syllable type, and *r* the probability to repeat a syllable type). With these functions it is thus not possible to describe cases where a higher repetition rate is more likely than a lower one.

The occurrence of syllable type doublets were quite well represented by the MM. But this was not the case for the syllable type triplets. First-order MMs only use the transition probabilities between two consecutive states, not the transition probabilities between three or more consecutive states. As our first-order MM could not explain the occurrence of triplets, we conclude that the song of mice follows a higher-order sequencing, in which one syllable type does not only depend on its directly preceding syllable type, but e.g. also the pre-preceding syllable type. These results support the study of Holy and Guo [3] on the USV of male inbred mice, who used only two syllable types (with or without one or more jumps). However, to disentangle the complex sequencing of syllables in mouse USV in more detail, it will very likely be necessary to use a larger number of syllable types, better representing the differences in the syllables. The next steps in this analysis will be to check more complex models like e.g. Hidden Markov models [61], which are often used to model the sequences underlying human languages and genomes [61], [62].

We also compared the usages of syllable types between populations and sexes. As described above, German mice used more turns, French mice more jumps. Female mice used more jumps, male mice more upward modulated syllables. The start and stop syllables were rather similarly distributed like the general usage. However, both populations and sexes used less jump syllables to start or to stop a song.

We conclude that wild house mice do not sequence their syllable types randomly on their prevalence or single transition probabilities, but follow a more complex temporal sequencing system which can be called a syntax. Such a syntax could evidently convey some information that can be interpreted by a receiver. Hoffmann et al. [38] have suggested that male mice could signal individuality and kinship to others, based on canonical discriminant analysis of song parameters. In our experiment, we see also females emitting complex songs, implying that there is a two-way signalling of information between the sexes, but also in same sex interactions.

### USV are part of a complex communication system

A complex communication system is expected to arise in systems of social complexity [23]. We found indeed in our previous semi-natural environment experiments, using the same populations, that complex extended family structures arise, including multiple mating with kin and relatives, but also pair bonding over extended times [25]. Furthermore, it is known that house mice engage in communal nesting, which requires also a higher level social organization [53], [54]. Even interactions that could be described as empathy appear to occur frequently [63]. Hence, the communicative complexity reflects very well the social complexity in house mice. Freeberg et al. [23] have stated in their review: "Units in which a greater number of distinct social roles exist represent greater social complexity than units with very few distinct social roles. For example, one group might contain post-reproductive females and males, reproductive females and males, reproductively mature but non-breeding young adults, sets of offspring from a previous breeding season, and sets of current offspring, whereas another group might contain reproductive females and males and their sets of current offspring. The former group would represent more of an ‘information centre’ and more of a unit of collective, adaptive behaviour because of its diversity of social roles, compared with the latter group. As such, we would predict greater complexity of communicative signals in units with more distinct social roles compared with those with fewer distinct social roles." This description of a complex social group is fully in line with the situation we find for wild mice living under semi-natural conditions [25]. Hence our inferences on communication complexity match well with this situation and support the prediction made by Freeberg et al. [23].

### USV might play a role in population divergence

Given that experiments with knockout mice for hearing [11] as well as cross-fostering experiments [12] have suggested that USV patterns are mostly genetically determined, one would have to look for genetic circuits that have changed between the populations to explain the above described divergence of song parameters between the populations. The *Cntnap2* gene pointed out in the introduction is such a gene: it is known to be involved in vocalisation phenotypes and it indeed shows a high differentiation between the populations. In fact, the differentiation is caused by a selective sweep in the German population (suppl. Figure S1) which would imply that an adaptive process was involved in generating the divergence. This could be sexual selection or the adaption to ecological factors like predator avoidance or habitat-dependent sound transmission properties as it was shown for birds [64]. However, the ecological factors between our two populations do not differ much, i.e. the influence of these factors on French and German USV can likely be neglected. Sexual selection, on the other hand, would imply a co-evolution of preferences and signals, which could be an active process leading to divergence over time. However, sexual selection is usually only considered to influence mating signals, while the divergence that we see includes also the social communication between same sex partners. This could be either a pleiotropic by-product of selective divergence of mating signals, or a process akin to sexual selection is also relevant for social communication, namely co-evolution between signals and receivers. This would be of particular importance in social systems of high complexity [23], as discussed above. Interestingly, *Cntnap2* is also among the *FoxP2* target genes that show adaptive differentiation in human populations [65], where the same principles might apply.

## Conclusions

While studies of USVs in inbred mouse strains have laid important foundations for exploring basic principles of ultrasonic communication, it is clear that studies in wild mice and under semi-natural conditions can provide new insights into the role and the evolution of USVs. Our results add two new aspects that need to be further explored. One is the role of USVs in social communication, outside of mating contexts. The other is the complexity of the syntax that may convey information and may include a learning component that has so far not been much considered (but see [13]). Finally, the complexity of USVs in mouse populations appears to support the social complexity hypothesis as a factor in communicative complexity [23].

## Supporting Information

Figure S1 **Selective sweep in the first intron of the Cntnap2 gene.** The data are taken from Staubach et al. 2012 [29]. The figure shows the UCSC genome browser tracks of the region, whereby each line represents one haplotype of the respective population. Blue and red vertical lines represent the SNP polymorphisms (connected by horizontal bars of the corresponding colour) (see Staubach et al. 2012 for further details). The region in the yellow box shows the sweep region, as identified by the Rsbl and the XPCLR statistics (Staubach et al. 2012). The gene structure is shown in the thin blue line below. The sweep region covers mostly the first intron of the gene, which corresponds to the region where FoxP2 is expected to bind (inferred from the corresponding data in humans (Vernes et al. 2008)). It is therefore likely that the sweep is caused by a change in the regulatory interaction between FoxP2 and Cntnap2.
(TIF)

Figure S2 **Dichotomous distribution of sound recordings.** The frequency distribution of all vocalisations that have been recorded plotted as the minimum vs. maximum frequency of each vocalisation. The distribution of vocalisations shows two main clusters. The few vocalisation contained in the cluster around 20 kHz are not part of the typical USVs of wild mice described in the literature [37], [38]. They were thus excluded from further analysis.
(TIF)
