# Correction: A Role for Ultrasonic Vocalisation in Social Communication and Divergence of Natural Populations of the House Mouse (*Mus musculus domesticus*)

**DOI:** 10.1371/journal.pone.0118130

**Published:** 2015-01-30

**Authors:** The PLOS ONE Staff

There is an error in the fifth row values for Table 1. Please see the correct Table 1 here.

**Table 1:** All temporal and spectral parameters used in the main analysis. For each group of mice the mean (+/- standard deviation) is given.

****	**German females**	**German males**	**French females**	**French males**
Quantitative parameter set 1: number of songs				
songs/night	20.3 (+/- 14.5)	12.53 (+/- 13.74)	17.2 (+/- 14.3)	14.6 (+/- 45.8)
Quantitative parameter set 2: temporal data				
song duration	505.9 (+/- 382.9)	300.3 (+/- 181.0)	565.3 (+/- 479.4)	294.0 (+/- 183.4)
syllables/second	6.99 (+/-1.91)	8.55 (+/-3.44)	7.62 (+/-2.37)	8.62 (+/-3.37)
Qualitative parameter set: syllable data				
duration	69.4 (+/- 36.5)	46.3 (+/- 36.5)	50.6 (+/- 36.4)	36.5 (+/- 26.0)
freqsta(1)	78.4 (+/- 8.2)	80.1 (+/- 14.2)	76.3 (+/- 10.8)	81.5 (+/- 18.6)
freqslope(1)	0.1 (+/- 0.3)	0.2 (+/- 0.5)	0.1 (+/- 0.5)	0.1 (+/- 0.6)
freqmin(1)	68.6 (+/- 11.4)	75.0 (+/- 14.0)	65.3 (+/- 11.1)	75.5 (+/- 18.5)
freqband(1)	23.0 (+/- 14.4)	14.5 (+/- 10.6)	22.9 (+/- 14.3)	15.1 (+/- 14.4)
freqCOG(1)	78.1 (+/- 10.6)	82.6 (+/- 13.3)	75.6 (+/- 9.8)	83.6 (+/- 18.8)
jumps	0.6 (+/- 0.9)	0.1 (+/- 0.4)	0.8 (+/- 1.1)	0.2 (+/- 0.5)
turns	2.0 (+/- 1.8)	1.2 (+/- 1.5)	2.0 (+/- 2.2)	0.9 (+/- 1.1)

(1) start frequency, frequency slope (calculated as change of frequency in kHz per ms), minimum frequency, frequency band (calculated as change of frequency in kHz per ms) and COG of frequency (COG = Centre of gravity, see Methods section Sound analysis for calculation).

Additionally, there is an error in the values for the parameter syllables/second for Fig. 5b. Please view the correct Fig. 5 here.

**Fig 5:**
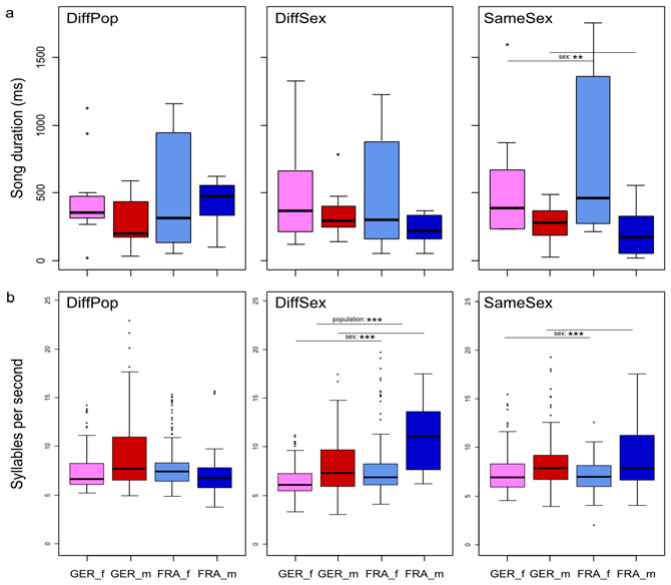
Song duration (a) and syllables rate (b) in the different social contexts. Box plots are separated by sex and population. Abbreviations and colours as in Figure 4. Asterisks denote the cases where found differences were significant (p≤0.05 (*), p≤0.01 (**)).

Finally, there is an error in the last two sentences of the last paragraph of the “Temporal Analysis” sub-section under the “Results” section. Please refer to the correct paragraph here.

To find the factors responsible for these differences, we conducted post-hoc tests (Wilcoxon rank-sum test) for the DiffSex and the SameSex situations and corrected for multiple testing (Bonferroni, DiffSex: p’ = p * 4, SameSex: p’ = p * 2). In the SameSex situation females emitted longer songs than males (W = 245, p’ = 0.0085). In the DiffSex situation French mice emitted more syllables per second than German mice (W = 27820, p’ < 0. 0001). In both situations, males emitted more syllables per second than females of the same population (DiffSex: W = 27029, p’ < 0.0001; SameSex: W = 14304, p’ < 0.0001).
