# Supplementary figures and images for: A Role for Ultrasonic Vocalisation in Social Communication and Divergence of Natural Populations of the House Mouse (Mus musculus domesticus)
Source: PLoS One. 2014 May 9;9(5):e97244. doi: 10.1371/journal.pone.0097244 (PMC4016290; doi:10.1371/journal.pone.0097244)

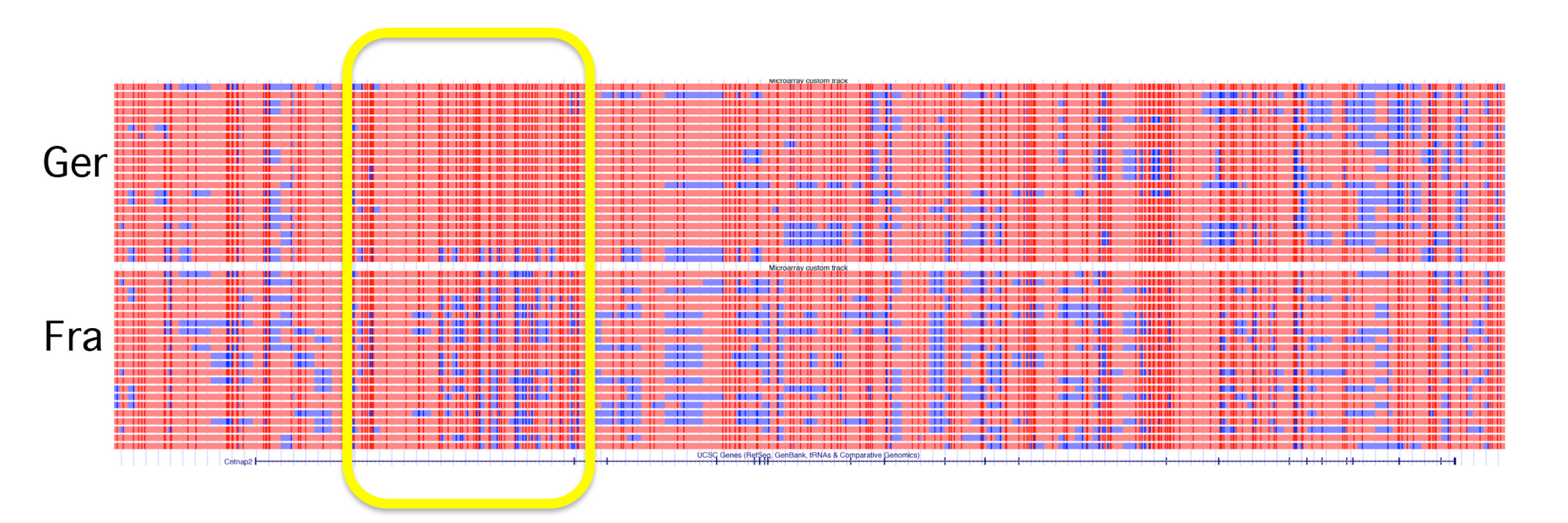

Supplement: Figure S1 — Selective sweep in the first intron of the Cntnap2 gene. The data are taken from Staubach et al. 2012 [29]. The figure shows the UCSC genome browser tracks of the region, whereby each line represents one haplotype of the respective population. Blue and red vertical lines represent the SNP polymorphisms (connected by horizontal bars of the corresponding colour) (see Staubach et al. 2012 for further details). The region in the yellow box shows the sweep region, as identified by the Rsbl and the XPCLR statistics (Staubach et al. 2012). The gene structure is shown in the thin blue line below. The sweep region covers mostly the first intron of the gene, which corresponds to the region where FoxP2 is expected to bind (inferred from the corresponding data in humans (Vernes et al. 2008)). It is therefore likely that the sweep is caused by a change in the regulatory interaction between FoxP2 and Cntnap2. (TIF) [file pone.0097244.s001.tif]

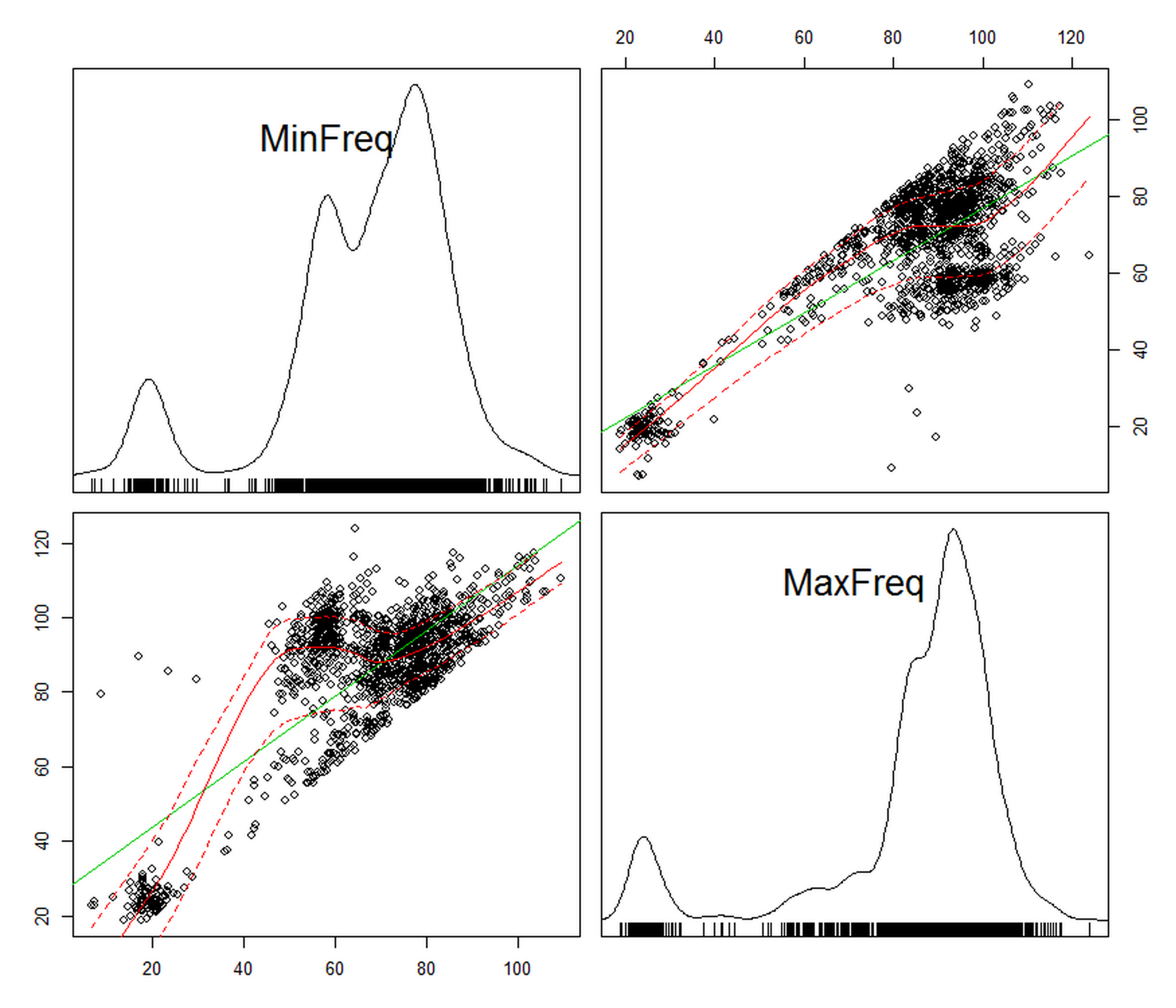

Supplement: Figure S2 — Dichotomous distribution of sound recordings. The frequency distribution of all vocalisations that have been recorded plotted as the minimum vs. maximum frequency of each vocalisation. The distribution of vocalisations shows two main clusters. The few vocalisation contained in the cluster around 20 kHz are not part of the typical USVs of wild mice described in the literature [37], [38]. They were thus excluded from further analysis. (TIF) [file pone.0097244.s002.tif]
